# An Attempt to Quantitate “Value” In Medical Oncologic Therapy

**DOI:** 10.7759/cureus.2810

**Published:** 2018-06-14

**Authors:** Christie Hancock, Linda Green, Timothy Lestingi, Jacob Bitran, MD

**Affiliations:** 1 Medicine, Advocate Lutheran General Hospital, Park Ridge, USA; 2 Radiation Oncology, Advocate Lutheran General Hospital, Park Ridge, USA; 3 Oncology, Advocate Lutheran General Hospital, Park Ridge, USA

**Keywords:** incremental cost effectiveness ratio, value, chemotherapuetics

## Abstract

Objective

We wanted to examine the incremental cost-effective ratio (ICER) for a variety of Food and Drug Administration (FDA) approved oncology drugs in the adjuvant or curative setting to determine the value provided.

Design

We examined the annualized incremental drug costs of a variety of FDA approved chemotherapeutic drugs used in an adjuvant or curative setting based on National Comprehensive Cancer Network (NCCN) category 1 practice guidelines for melanoma, Her2/neu over-expressive breast cancer, renal cell carcinoma, stage IIIA non-small cell lung cancer, myeloma, B cell lymphoma, and Hodgkin lymphoma. The studies we examined were randomized clinical trials on which the NCCN guidelines are based; we solely examined the incremental cost-effectiveness of the trial drug as we assumed that the costs of the health care provided were equivalent between the two treatment arms. We used a formula to determine the incremental cost-effectiveness ratio (ICER). The ICER compares a new intervention (C new) with its alternate (C alt) divided by the quality-adjusted life-years (QALY) that results from the new intervention (QALY new) versus the alternate (QALY alt) and is expressed as ICER = (C new-C alt)/(QALY new-QALY alt). The QALY’s were derived from what was reported in the study and based on the incremental disease-free survival.

Results

Drugs such as rituximab provide high value in the curative therapy for lymphoma. Drugs such as adjuvant dabrafenib and trametinib provide intermediate value in the treatment of melanoma, and similarly with maintenance lenalidomide in myeloma and adjuvant trastuzumab in breast cancer. Oncologic drugs that provide low value include adjuvant ipilimumab in melanoma, adjuvant sunitinib in renal cell carcinoma, adjuvant neratinib in breast cancer, adjuvant durvalumab in lung cancer, and brentuximab in the curative therapy for Hodgkin’s lymphoma.

Conclusion

The ICER needs to be evaluated for newly approved FDA oncology chemotherapeutic drugs before incorporating them into routine clinical practice.

## Introduction

The word 'value' is bantered about in healthcare; however, definitions of value are often nebulous at best. Porter [1] defines value in healthcare as outcomes achieved per dollars spent. Newmann et.al. [2} promote using cost-effective analysis (CEA) as a means of defining the value of public health interventions. Furthermore, they define cost-effectiveness thresholds such as $50,000 to $100,000 per quality-adjusted life-years (QALY). The World Health Organization (WHO) has proposed a cost-effectiveness threshold based on a country’s gross domestic product (GDP). They suggest a benchmark of three times the GDP per capita as the upper threshold for an acceptable level of cost-effectiveness [3]. Anderson et al. [4] have proposed the integration of a value benchmark in clinical guideline recommendations in the field of cardiology. High-value outcomes are defined as less than $50,000 per QALY gained; intermediate value is greater than $50,000 but less than $150,000 per QALY gained. Low-value interventions are those that exceed $150,000 per QALY gained. The incremental cost-effectiveness ratio (ICER) is a means of comparing an intervention or program with the best alternative or standard of care divided by the QALY gained and can be expressed as ICER = C new-C alternate/QALY new-QALY alternate, where C is the cost of therapy or intervention and QALY represents the additional years or months gained [2, 4].

The American Society of Clinical Oncology (ASCO) issued a position paper aimed at “contributing to the national dialogue on rising cancer drug prices” [5]. According to the position paper, “while new classes of drugs have achieved unprecedented success in a growing number of cancers, in some cases the price of a new drug bears no relationship to its effectiveness” [5]. According to one study, only 19% of cancer drugs approved by the Food and Drug Administration (FDA) produced clinically meaningful outcomes in patients despite their high prices [6]. This position paper advocates “that the FDA use clinically meaningful outcomes for patients when assessing new and supplemental drug applications, rather than small benefits that achieve statistical significance in large trials” [5]. They advocate Medicare test “a value-based pathway” to incentivize both providers and the pharmaceutical industry to develop “high-value” treatments.

We aim to examine the ICER of approved FDA oncology drugs using the above formula. We believe that the greatest impact of cancer therapy is in either the adjuvant or curative setting, as it may ultimately translate into longer disease-free survival, which is tantamount to cure. Therefore, we restricted our analysis to the adjuvant or curative context rather than simple palliation. The focus of this study is to help determine annual incremental costs and whether a given therapy has high, intermediate, or low value [4].

## Materials and methods

Patient and public involvement

Patients were not involved in the conduct of this study.

We used National Comprehensive Cancer Network (NCCN) practice guidelines as the basis of our analysis [7]. In the instance of Hodgkin's lymphoma, the 2018 version is being drafted. We reviewed the phase III randomized clinical trials that formed the basis of the NCCN category-1 evidence-based recommendations “where there is uniform consensus that intervention is appropriate” [7]. We limited our search to trials in the adjuvant or curative setting where an intervention carries the greatest impact; moreover, we restricted our analysis to trials that employ drugs that cost more than $5000 per month. The analysis was restricted to adjuvant trials in melanoma, Her2/neu positive breast cancer, renal cell carcinoma, and stage IIIA non-small cell lung cancer. The curative trials were limited to diffuse large B cell lymphoma, mantle zone lymphoma, myeloma [7], and a newly published study on Hodgkin's lymphoma [8]. In reviewing the trials that formed the basis of NCCN practice guidelines, we quantitated “value” based on incremental per patient costs by using the formula ICER = C new-C alternate/QALY new-QALY alternate [2, 4]. Clearly, if a drug or molecule can be used that leads to a longer disease-free survival in either the adjuvant or curative setting at what is defined as a reasonable annualized price of less than $150,000, it provides value [4].

The randomized studies chosen were three adjuvant melanoma trials [9-11], three trials examining the role of maintenance lenalidomide in myeloma [12-14], two trials testing rituximab in a curative or post autologous bone marrow transplant setting [15-16], two studies examining adjuvant trastuzumab in Her2/neu over-expressive breast cancer [17-18], one trial examining adjuvant neratinib in Her2/neu over-expressive breast cancer [19], one study testing the role of brentuximab when substituted for bleomycin in the curative treatment of Hodgkin’s lymphoma [8] one study examining adjuvant sunatinib in renal cell carcinoma [20], and single trial of adjuvant durvalumab in stage IIIA non-small cell lung cancer [21].  We assumed that the costs of providing healthcare would be equivalent between the randomized arms, and thus we simply focused on the incremental costs associated with the trial drug. The costs of drug administration were based on 2018 drug pricing as shown in Table 1. Since many of the studies did not report quality of life or overall survival, we could not quantitate QALYs as normally defined. All studies did report disease-free survival, and this endpoint was used as the QALY measurement and is expressed in years.

**Table 1:** 2018 Pricing of Chemotherapeutic Drugs

Drug	Price
Brentuximab	96.80/mg
Dabrafenib	9929.00/mo
Durvalumab	15000.00/mo
Ipilimumab	157.46/mg
Lenolidamide	14194.70/mo
Neratinib	1700.00/mo
Nivolumab	28.78/mg
Sunitinib	15027.14/28 caps
Trametinib	11265.07/mo
Trastuzumab	5222.89/440mg

## Results

The results are shown in Table 2.

**Table 2:** Annualized Incremental Cost Effectiveness Ratios (ICER) for Various Chemotherapeutic Drugs AC-T: Doxorubicin, cyclophosphamide, paclitaxel CHOP: Cyclophosphamide, doxorubicin, vincristine,  prednisone ABMT: Autologous bone marrow transplantation ABVD: Doxorubicin, bleomycin, vincristine, DTIC A+AVD: Brentuximab + doxorubicin, vincristine, DTIC D+T: Dabrafenib + trametinib Nivo: Nivolumab Ipi: Ipilimumab L: Lenalidomide P: Placebo M+P: Melphalan and prednisone D: Darvalumab N: Neratinib

Reference	Treatment Arms	ICER formula	Annualized ICER
#9	Stage III =/- Ipi	$1763552/yr	$1,763,552
#10	Stage III BRAF + D + T vs P	$254828/2.83 yr	$90,046
#11	Stages III/IV Nivo vs Ipi	($145051)2.32 yr	($62,608)
#12	ABMT +/- L	$1703361/1.58 yr	$107,808
#13	ABMT +/- L	$1703361/1.5 yr	$113,557
#14	M+P +/- L	$1703361/1.5 yr	$113,557
#17	AC-T +/- trastuzumab (H)	$54000/.44 yr	$122,727
#18	Chemotherapy +/- H	54000/.2 yr	$270,000
#19	Chemotherapy & H+/- N	$120871/.16 yr	$755,444
#20	Surgery +/- sunitinib	$301259/1.2 yr	$251,049
#21	Chemoradiotheray +/- D	$180000/.93 yr	$193,548
#15	CHOP +/- rituxan	$20100/2 yr	$10,500
#16	ABMT +/- rituxan	$60300/1 yr	$60,300
#8	A+AVD vs ABVD	$97540/.1 yr	$975,740

As shown in Table 2, we conclude that rituximab provides high value in the treatment of diffuse large B cell lymphoma and when used in the setting of post-transplant therapy for mantle zone lymphoma. Similarly, trastuzumab, used as prescribed by Romond [17] in the adjuvant treatment of Her2/neu positive breast cancer, provides intermediate “value” with higher cure rates. However, giving trastuzumab for two years rather than one leads to incremental costs without additional benefit [18] and is thus of low value. Using neratinib for a year following trastuzumab-based adjuvant chemotherapy for Her2/neu positive breast cancer is of low value [19]. Using sunitinb as adjuvant therapy in the treatment of high-risk renal cell carcinoma based on current US pricing is of low value [20]. Lenalidomide provides intermediate value when used as maintenance therapy following autologous bone marrow transplantation (ABMT) in the treatment of myeloma or following melphalan and prednisone for myeloma patients who are not candidates for ABMT [12-14]. In the context of adjuvant therapy for melanoma, ipilimumab as prescribed by Eggermont is cost prohibitive and of low value [9]. In contrast, adjuvant nivolumab is significantly less expensive than ipilimumab and provides intermediate value [10]. Adjuvant dabrafenib and trametinib in the treatment of BRAF-mutated melanoma provides intermediate value [11]. In the case of Hodgkin’s lymphoma, using brentuximab vedotin in lieu of bleomycin when given with doxorubicin, vinblastine, and DTIC is of low value [8]. Similarly, administering durvalumab following chemoradiotherapy in stage III non-small cell lung cancer is of low value [21].

## Discussion

The costs of cancer care are escalating annually. Smith and Hillner predict that by 2020 the annual direct costs of cancer care will exceed $173 billion [22] as escalating costs are driven by new drugs, many of which are priced at $5000 per month or more [23]. Recently, the FDA approved tisagenleceucel a chimeric antigen receptor T cell (CAR-T) for the treatment of pediatric and young adult patients with acute lymphoblastic leukemia (ALL). The CAR-T therapy is delivered as a one-time infusion at a cost estimated to be $475,000 [23]. There is no doubting the effectiveness of this therapy; however, the cost is high [23]. Bach, Giratt and Saltz state “the price of $475,000.00 for a single treatment with tisagenleceucel is difficult to put into context because expensive cancer treatments typically cost $10,000.00 to $20,000.00 per month and reflect prices that already increased more rapidly than the benefits they have provided patients over the past decades" [23].

We examined the ICER of various oncology drugs, some of which are already FDA approved, have become standard of care, and are incorporated into clinical oncology practice. We used the standard formula for ICER and determined annualized costs. We categorized these oncology drugs according to value as proposed by Anderson et.al. [4]. Some drugs such as rituximab provide high value. Others such as trastuzumab, lenalidomide, nivolumab provide intermediate value (Table 2). Still others provide low value and are clearly not cost-effective (Table 2). The case of neratinib is of particular interest. Neratinib in our analysis provides low value when used as an adjuvant therapy following trastuzumab in women with Her2/neu over-expressive breast cancer. The FDA approved neratinib’s use as an adjuvant therapy on July 17, 2017 [24]. Despite FDA approval, Dr Steven Vogl, a breast cancer expert, is quoted as saying “Should we recommend extended adjuvant neratinib to patients? No! is the short answer.” Dr. Vogl’s opinion is based on the drug's slim benefits and the adverse toxicity profile [25]. Interestingly, the European Union panel refused to approve neratinib based on the very modest benefit provided [26]. Clinicians need to be cognizant of ICER when prescribing oncology drugs newly approved by FDA and while incorporating them into clinical practice.

The mission of the FDA is not to define value; rather, its responsibility is “protecting the public health by assuring safety, efficacy and security of the human and veterinary drugs, biologic products, medical devices, the nation’s food supply, cosmetics, and products that emit radiation…responsible for advancing the public health by helping to speed innovations that make medicines more effective, safer, and more affordable by helping the public get scientific based information” [27].  We acknowledge the scientific validity of the studies we cite and the objectivity that went into the FDA approval process. However, at a time in our country’s history when resources are strained, we contend that in addition to statistical significance, the clinical impact and value as defined by ICER needs to be considered before a new novel drug becomes incorporated into the “standard of care”.

## Conclusions

We contend that in addition to statistical significance, the clinical impact and the value of a drug as defined by ICER needs to be considered before a new drug becomes incorporated into the “standard of care”.
